# A Rare Case of T-Large Granular Lymphocytic (T-LGL) Leukemia in a Patient With Rheumatoid Arthritis With Neutropenia and Low LGL Level

**DOI:** 10.1155/crom/5013991

**Published:** 2025-08-15

**Authors:** Muhammad Daniyal, Anamm Polani, Pavel Bleik, Jeffrey Allerton

**Affiliations:** ^1^Department of Internal Medicine, Bassett Medical Center, Cooperstown, New York, USA; ^2^Department of Hematology and Medical Oncology, Bassett Medical Center, Cooperstown, New York, USA

## Abstract

T-large granular lymphocytic (T-LGL) leukemia is a rare hematological malignancy characterized by clonal expansion of cytotoxic T-cells resulting in cytopenias. The diagnostic criteria for T-LGL leukemia necessitated a sustained peripheral blood elevation of LGLs exceeding 2 × 10^9^/L for a minimum duration of 6 months, in the absence of an identifiable etiology. In most of the cases, it is associated with autoimmune disorders such as rheumatoid arthritis. As cytopenias, including neutropenia, can be an early manifestation of the disease, they may get confused with Felty's syndrome, resulting in delayed diagnosis and treatment. Hence, we are presenting a rare case of diagnosing T-LGL leukemia in a patient with rheumatoid arthritis with neutropenia and low LGL level.

## 1. Introduction

T-large granular lymphocytic (T-LGL) leukemia is a rare hematological malignancy, accounting for approximately 2%–5% of all cases of chronic lymphoproliferative diseases in the United States [1]. T-LGL is characterized by a predominantly indolent clinical course in most cases, presenting with elevated peripheral blood levels of large granular lymphocytes (LGLs), cytopenias, and splenomegaly while typically lacking lymphadenopathy. Traditionally, the diagnostic criteria for T-LGL leukemia necessitated a sustained peripheral blood elevation of LGLs exceeding 2 × 10^9^/L for a minimum duration of 6 months, in the absence of an identifiable etiology. A distinctive hallmark of T-LGL leukemia is its intriguing correlation with autoimmune disorders. Notably, rheumatoid arthritis (RA) emerges as a prevalent comorbidity, affecting approximately 17%–28% of T-LGL leukemia patients (referred to as RA-associated T-LGL leukemia).

The updated criteria for diagnosing T-LGL leukemia allow for diagnosis with a clonal T-cell population in patients with autoimmune diseases or cytopenias, irrespective of the LGL count or waiting period. Particularly, the diagnosis of T-LGL leukemia can be diagnostically challenging in cases presenting with a low LGL count, which represents a nonleukemic variant of the disease, accompanied by pronounced splenomegaly. This complexity is further compounded when T-LGL leukemia occurs in patients with concurrent RA, necessitating careful differentiation from Felty's syndrome (FS) and hepatosplenic T-cell lymphoma (HSTCL) in the context of RA [2].

Of note, FS is a rare subset of RA characterized by neutropenia and splenomegaly. Clinically, distinguishing cases of RA-associated T-LGL leukemia without absolute lymphocytosis in peripheral blood but with concomitant neutropenia from FS can be challenging. Misinterpretation of neutropenia and splenomegaly as FS may lead to overlooking these cases. However, differentiation between RA-associated T-LGL leukemia and FS can be accomplished through the assessment of T-cell receptor (TCR) gene rearrangement. Monoclonal TCR gene rearrangements, indicating T-cell clonality, are present in T-LGL leukemia but absent in FS [3]. Therefore, we would like to describe a rare case of T-LGL leukemia in a patient with RA with neutropenia and low LGL level.

## 2. Case Presentation

A 62-year-old female was hospitalized in the summer of 2021 for fever and generalized arthralgia (mostly bilateral hands, knees, and wrists) and was found to have elevated ESR/CRP, pancytopenia, and elevated RF of 127 IU/mL (ref. range < 14 IU/mL), CCP of 251 U (ref. range < 20 U), and ANA of 1:160 consistent with a homogeneous pattern.

**Figure 1:**
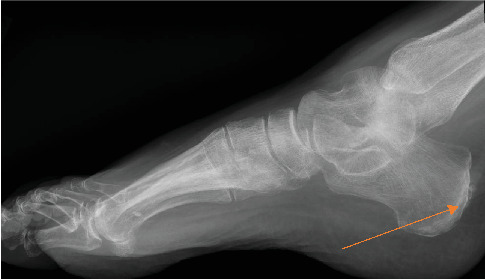
X-ray of the left foot demonstrating degenerative changes and mild retrocalcaneal spur (orange arrow).

Her presentation was initially thought to be from tick-borne illness despite negative testing for *Babesia*, *Anaplasma*, and *Ehrlichia* and was treated with doxycycline, with persistent pancytopenia. She was seen in the hematology clinic for the latter, in the setting of elevated inflammatory markers and RF, and ACD (anemia of chronic disease). The patient was started on filgrastim and steroids for autoimmune neutropenia thought to be from possible RA given she had complaints about joint stiffness and pain in the morning, elevated inflammatory markers (ESR/CRP), and rheumatoid factor with CCP, as well as an x-ray of the foot demonstrating degenerative changes and a retrocalcaneal spur, which is nonspecific but seen in RA (Figure 1).

**Figure 2:**
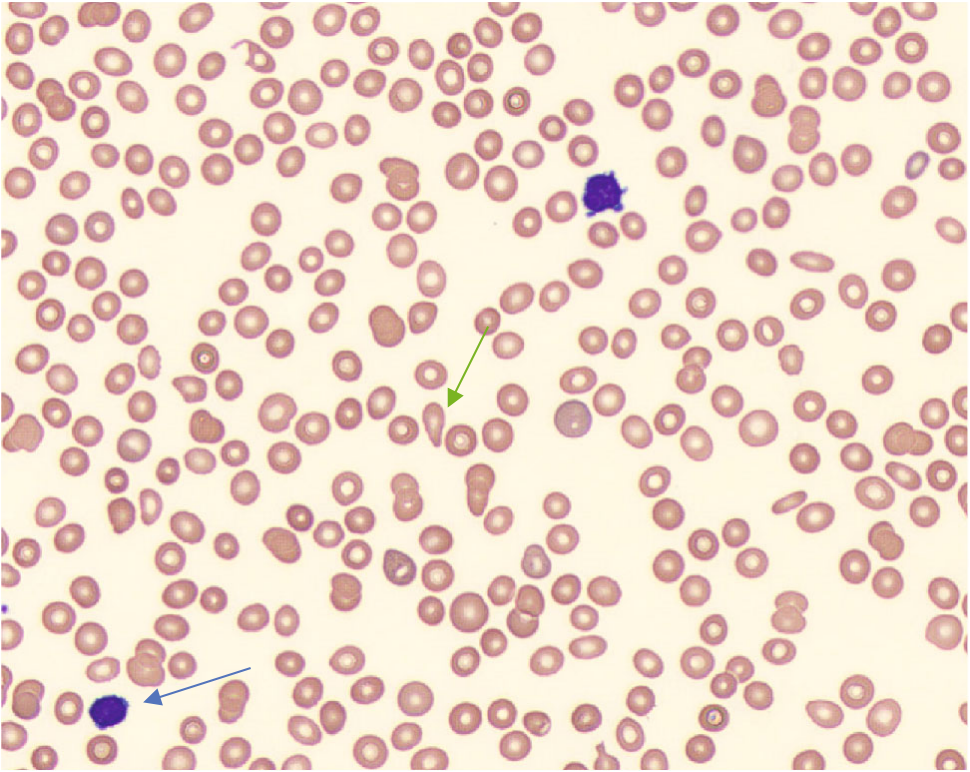
Initial blood smear. Blast cell is seen (blue arrow), along with tear drop cells (green arrow).

Upon initial presentation, her complete blood count was as follows: WBC of 1.7 × 10e3 cells/*μ*L (ref. range 3.7–10.6e3 cells/*μ*L), normal RBC count of 4.25 × 10e6 cells/*μ*L (ref. range 3.7–5.10e6 cells/*μ*L), hemoglobin of 11.2 (ref. range 11.5–15.5 g/dL), MCV of 79.3 fL (ref. range 81.0–99.0 fL), MCH of 26.3 pg (ref. range 27–33.5 pg), and platelet count of 101 × 10e3 cells/*μ*L (ref. range 140‐425 × 10e3 cells/*μ*L). Her differential was as follows: ANC of 850 cells/*μ*L (ref. range 1500–7400 cells/*μ*L), ALC of 510 cells/*μ*L (ref. range 950–3500 cells/*μ*L), and monocyte of 20% (ref. range 2.0%–14.0%) along with blast count of 1.0% (normal = 0%). Her blood smear did reveal evidence of blast cell presence, some discoid-shaped and polychromatic RBC, along with evidence of tear-drop cells (Figure 2). Her total LGL population was 2% (ref. range < 6%).

She also underwent a bone marrow biopsy, which did not reveal any clonal aberrations and was consistent with a reactive process possibly from the underlying inflammatory disorder. A total of 20 metaphase spreads were analyzed by G-banding.

No NK activity was identified on bone marrow flow cytometry, which overall demonstrated a normal B-cell population with a marked excess of activated CD8+ T-cells, best resolved by dimmed CD3 and CD5. CD45/CD10 gating confirmed a relative paucity of mature granulocytes.

Patient had slightly improved neutrophil count with oral steroid (prednisone) and was started on methotrexate 10 mg weekly in September 2021, with the goal to taper off steroids to prevent long-term steroid use. After around 3 months, while the patient had slight transient improvement in neutrophil count with methotrexate, her ANC remained in the range of 209–275, for which weekly filgrastim was added and MTX dose was increased to 20 mg weekly for the goal of ANC > 500. She remained on the regimen until the summer of 2022, when she started developing intolerance to MTX and had worsening neutropenia (ANC now below 100), persistent unintentional weight loss, and worsening arthritis. MTX was then discontinued, and she was started back on steroids and TNF inhibitor, adalimumab, with the goal to taper steroids once ANC becomes stable on the TNF inhibitor, treating for possible FA in the setting of autoimmune neutropenia, seropositive RA, and moderate splenomegaly/hepatomegaly on US abdomen (completed in the summer of 2022).

After a month of starting adalimumab, she developed low-grade fever, and in the setting of neutropenia and now low-grade fevers, HLH (hemophagocytic lymphohistiocytosis) workup was ordered by rheumatology showing elevated ferritin level of 321 ng/mL (normal range 16–243), triglycerides of 346 mg/dL (normal range < 150), and IL2-receptor of 1739*μ*/mL (normal range 122–496).

Patient was later seen in the hematology clinic and was noticed to have leukopenia with WBC 0.7 × 10e3 cells/*μ*L (normal range 3.7–10.6) and ANC 27 cells/*μ*L (normal range 1.500–7.400) along with thrombocytopenia with a platelet count of 17 × 10e3 cells/*μ*L. She was advised to be admitted to the hospital with concern of ITP flare likely due to tapering off her steroids.

During the hospitalization, she underwent imaging of her abdomen and pelvis, which now showed a massively enlarged spleen of up to 20.7 cm, portal vein dilation suggestive of portal HTN, and periportal lymphadenopathy. She was started back on high-dose steroids (1 mg/kg) with a slow taper, along with IVIG for ITP. Her filgrastim was also discontinued given massive splenomegaly and the risk of splenic infarct/rupture seen uncommonly secondary to filgrastim. A repeat bone marrow biopsy was obtained during hospitalization due to new lymphadenopathy, slightly rising LGL of 4%, poor response to MTX/steroids in the past, and worsening splenomegaly, raising concern for T-LGL, NK cell LGL leukemia, and HLH.

Her bone marrow biopsy this time was slightly different: TCR gamma PCR demonstrated evidence of a clonal population of cells with the same size rearrangement of the T-cell receptor gamma gene (TCRG) within a polyclonal T-cell background, consistent with lymphoid malignancy, but did not provide diagnostic value itself. Flow cytometry was also repeated, which demonstrated 39% of total events, of which 3% were CD19/20 positive B-cells with a kappa/lambda ratio of 2.1, 97% were CD3+ T-cells with a CD4/CD8 ratio of 0.2, and < 1% were CD3−/CD7+ NK cells. An aberrant population of CD5(dim) T-cells comprising 66% of lymphocytes and 18% of total cells was present. The overall population expressed CD3, CD7, CD2, and CD8 and was negative for CD4, TCR gamma–delta, and CD56, and no monotypic B-population was identified.

Overall the result of the biopsy came back favoring a CD8 positive T-cell neoplasm with a positive TCRG rearrangement, highly suggestive of T-LGL. NGS myeloid pattern was negative for STAT3 mutation. She was started on cyclophosphamide along with continuing steroids; however, adalimumab was discontinued.

Patient responded well to the combination of cyclophosphamide and steroids with improved anemia, neutropenia, and splenomegaly (16.8 cm on repeat CT 11/22, previously was 19.8 cm in 8/22).

## 3. Discussion

T-LGL leukemia is considered a rare disorder marked by the clonal expansion of cytotoxic lymphocytes, specifically cytotoxic T lymphocytes or natural killer cells. The precise pathophysiological mechanisms underlying T-LGL leukemia are still not fully understood. However, it is widely recognized that dysregulated signaling pathways and disrupted apoptotic pathways, likely influenced by chronic inflammation, contribute to the excessive proliferation of immune cells in this disease [4]. A notable observation in T-LGL leukemia is the frequent coexistence of an autoimmune disorder. In a specific study, approximately one-third of patients diagnosed with T-LGL leukemia were found to have RA as a concurrent condition [5]. The identification of neutropenia in RA patients should trigger further investigation. Evaluation of a peripheral blood smear represents a safe and noninvasive method to identify and quantify T-LGL cells, facilitating the diagnostic process [6].

A diagnosis of LGL leukemia is typically established when the peripheral blood LGL count exceeds 0.25 × 10^9^/L as per traditional view or 0.4‐0.5 × 10e3 cells/*μ*L as per currently accepted universal criteria, although most patients present with higher counts ranging from 2 to 10 × 10^9^/L. Subsequent assessment of bone marrow aspirate, accompanied by flow cytometry, is recommended to determine the T-LGL cell phenotype, providing valuable diagnostic guidance. In approximately 80%–90% of T-LGL leukemia cases, the phenotype of CD3(+), CD8(+), CD57(+), CD56(−), CD28(−), and TCR-*αβ*(+) is observed. Moreover, abdominal ultrasound findings indicative of splenomegaly can serve as additional supportive evidence for the diagnosis [7].

Of note, the occurrence of neutropenia in both T-LGL leukemia and FS is likely to involve a combination of cellular and humoral mechanisms. Cellular mechanisms are thought to play a prominent role by inhibiting the proliferation of neutrophil progenitors through intercellular interactions and the secretion of cytokines and chemokines. On the other hand, humoral mechanisms are believed to contribute primarily to survival defects. In the context of T-LGL leukemia and FS, these mechanisms may include direct destruction of myeloid precursors by infiltrating leukemic cells [8].

First-line treatment for T-LGL leukemia involves immunosuppressive therapy, such as methotrexate, cyclophosphamide, and cyclosporin A, targeting the underlying inflammation. If unresponsive, alternative chemotherapeutic or immunomodulatory agents may be used [9]. The hematologic management aims to reduce proinflammatory symptoms and prevent infections due to severe neutropenia. Monitoring response to therapy relies more on immunomodulatory effects rather than the number of T-LGL cells. Low weekly doses of methotrexate have shown a 50% remission rate in T-LGL leukemia cases, such as was discussed in a similar case of atypical aleukemic LGL-lymphocytic leukemia [10]. However, in our case report, the patient responded well to cyclophosphamide along with steroids.

## 4. Conclusion

T-LGL leukemia is a rare complication of advanced RA, and the presence of neutropenia in RA patients should be investigated further as it could be an early sign of T-LGL leukemia. Due to the limited number of T-LGL leukemia cases, it is important to increase awareness by publishing case reports that provide detailed information on diagnosis, treatment, and disease progression. This report is aimed at contributing to the existing literature by fulfilling this objective.

## Data Availability

The data that support the findings of this study are available from the corresponding author upon reasonable request.

## Nomenclature

ESR: erythrocyte sedimentation rate
CRP: C-reactive protein
CCP: cyclic citrullinated peptide
ANAs: antinuclear antibodies
NK: natural killer cells
MTX: methotrexate
ITP: immune thrombocytopenic purpura
HTN: hypertension
IVIG: intravenous immunoglobulin
CT: computer tomography
CD: cluster cell differential
TCR: T-cell receptor
ACD: anemia of chronic disease
WBCs: white blood cells
ANC: absolute neutrophil count
ALC: absolute neutrophil count
MCV: mean corpuscular volume
MCH: mean corpuscular hemoglobin
RBCs: red blood cells
LGL: large granular cell
HLH: hemophagocytic lymphohistiocytosis
FS: Felty's syndrome
